# A new method for acquiring long-term high-precision spatial data on rural settlements

**DOI:** 10.1016/j.mex.2021.101249

**Published:** 2021-01-26

**Authors:** Wei Song, Huanhuan Li

**Affiliations:** a Key Laboratory of Land Surface Pattern and Simulation, Institute of Geographic Sciences and Natural Resources Research, Chinese Academy of Sciences, Beijing 100101, China; b School of Land and Tourism, Luoyang Normal University, Luoyang 471000, China

**Keywords:** High precision rural settlement spatial data, KeyHole satellite images, ArcGIS, precision assessments

## Abstract

Accurate spatial data regarding rural settlements provides the basis for analyses of spatial evolution. Decrypted KeyHole military satellite images were used as a spatial data source in this study in order to assess historical rural settlements at high precision. The spatial data acquisition process used in this work involved three stages, interpretation from rural settlements, validation of results, and then resampling. We describe a new method to confirm validation samples in this article and use a confusion matrix to verify data accuracy. The Kappa value yielded in this approach was 0.97; the method described here can be used in the future for the acquisition of high-precision spatial data from historical rural settlements.

• Decrypted KeyHole military satellite images were used as a data source to obtain high-precision information regarding historical rural settlements.
• The new method proposed in this article was used to verify the accuracy of sample data and to solve the ongoing problem that historical image land use often cannot be verified.
• Result accuracy was verified using functions in ArcGIS 10.4 to create accuracy assessment points, update them, and compute a confusion matrix.

## Graphical abstract

**Figure:**
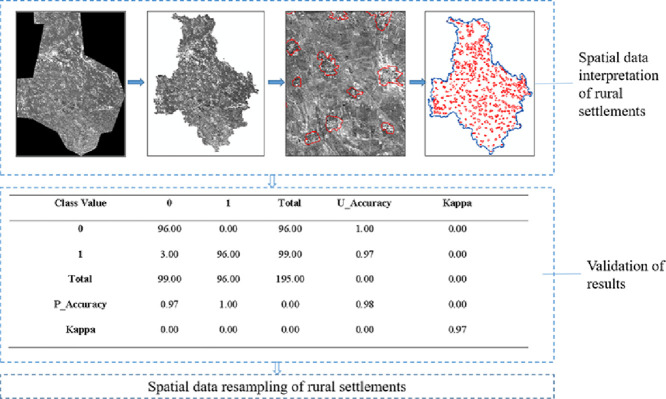

Specification table

**Table:** 

Subject area:	Earth and Planetary Sciences
More specific subject area:	Geography, land use, rural settlements
Method name:	Rural settlement identification using military satellite images
Name and reference of original method:	Song W., Li H., 2020. Spatial pattern evolution of rural settlements from 1961 to 2030 in Tongzhou District, China. Land Use Policy 105044.
Resource availability:	KeyHole satellite images: From Beijing Top View Technology Co., LTD. (http://www.topview.cc/); Land use map of 1980/2000/2015: remote sensing monitoring database of land use in China (http://www.resdc.cn/data.aspx?DATAID=184.)

## * Methodological details

The method proposed in this paper stems from our research on the evolution of spatial patterns and the simulated prediction of rural settlements across Tongzhou District, China, over the last 50 years [1]. Although accurate spatial data on rural settlements provides the basis for studying their spatial evolution, availability of this information remains limited. The bulk of previous studies have used TM (Thematic Mapper) image data [2], [3], [4] but the resolution of this resource (about 30 m) can only reveal the general characteristics of rural settlements. It therefore remains problematic to make clear judgments about internal structure using such data. A number of scholars have used QickBird, SPOT, and other image types to obtain rural settlement spatial data [5]. The cost of obtaining these images is relatively high, however, and so such sources are not widely used. Indeed, it is also the case that the bulk of these images were obtained after satellite technology became popular in the 1980s. This means that most surface information obtained from satellite images was collected over the last 30 years and so does provide a useful source of information with which to study the long-term evolution of rural settlements. Declassified military satellite images are used as data sources in this study to obtain historical high-precision spatial data from rural settlements.

The data acquisition process involved spatial data interpretation from rural settlements, result validation, and then resampling. The image correction benchmark used here for data interpretation and verification was 1980 land use status data. We used the functionality of ArcGIS 10.4 to create accurate evaluation points, to update these, and to calculate a confusion matrix to verify results. This paper proposes a method for obtaining historical rural settlement spatial data using decrypted military remote sensing (RS) satellite images as data sources and so can augment the research time limit to around 20 years. The method proposed in this paper will be useful for studying spatial distribution, historical evolution, and the internal structure of rural settlements.

The “rural settlements” referred to in this article comprise places where a population lived in a concentrated community. This means that land used includes homesteads built by rural residents, auxiliary land used for production and life, and villager public activity spaces.

## Data sources

We have studied the spatial evolution of rural settlements within Tongzhou District over the last 50 years [1]. On this basis, and taking study area characteristics and data acquisition into account, we selected spatial data on rural settlements within Tongzhou District in 2015, 2000, 1980, and 1961. Data sources for these different periods are shown in Table 1.

**Table 1:** Data sources and basic information about rural settlements in different periods.

Data	Data source	Brief descriptions
Rural settlement data for 1961	KeyHole satellite images Beijing Top View Technology Co., Ltd. (http://www.topview.cc/)	KeyHole is an American spy satellite series which is mainly used for military purposes. A total of around 930,000 RS images were captured by this series between 1959 and 1986. Images from first generation photo reconnaissance satellites have so been decrypted, including 860,000 satellite photos taken between 1960 and 1972; these were captured all over the world but are concentrated in Eastern Europe and Asia at a 2 m spatial resolution.
Rural settlement data for 1980/2000/2015	Land use map of 1980/2000/2015 Remote sensing database of land use in China (http://www.resdc.cn/data.aspx?DATAID=184)	Data were generated via artificial visual interpretation Landsat TM/ETM RS sensing at a 30 m spatial resolution.

## Data processing

### Rural settlement spatial data interpretation

Rural settlement data were extracted directly from the corresponding land use map for each of the years 1980, 2000, and 2015. Rural settlement data for 1961 were obtained from KeyHole satellite images.

**Fig. 1:**
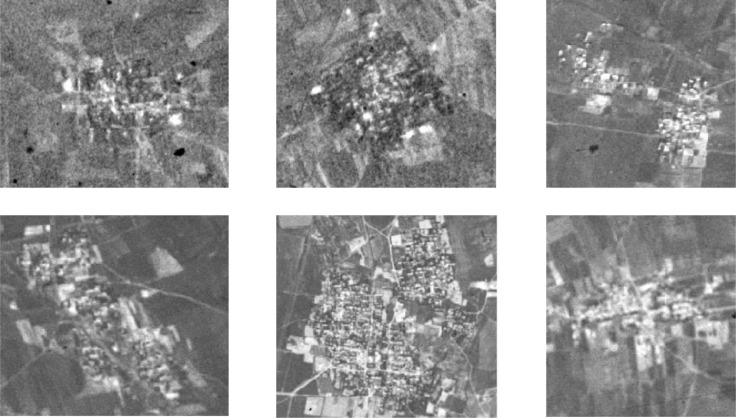
1961 KeyHole RS images of rural settlements.

**Fig. 2:**
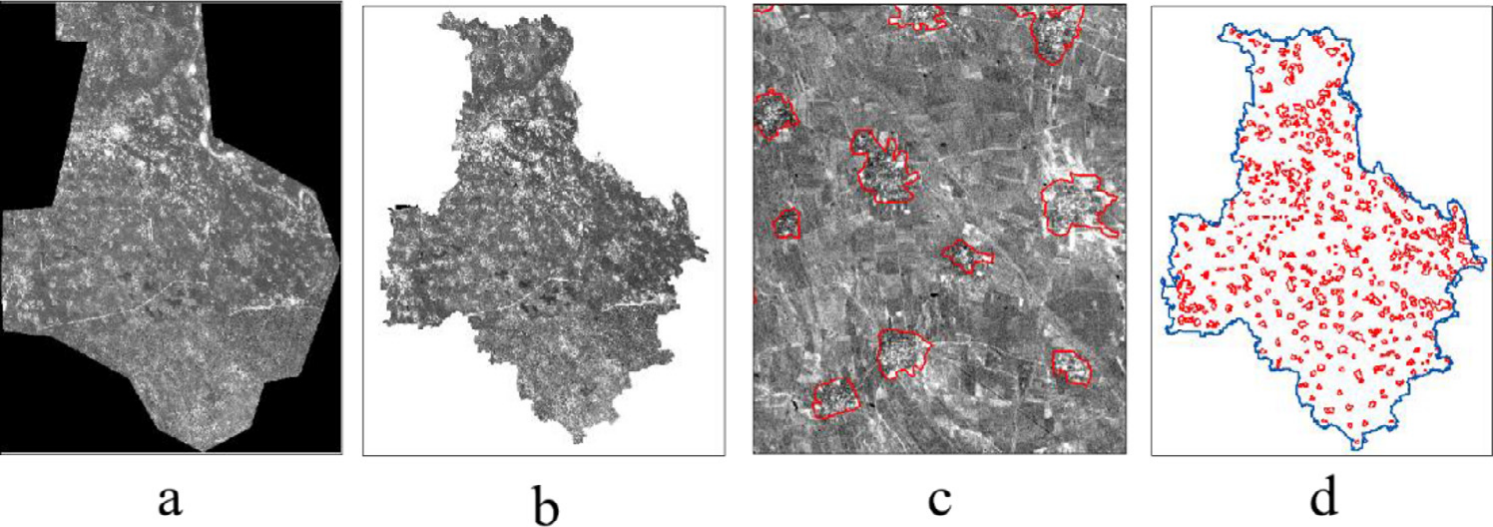
KeyHole RS image interpretations of rural settlements (a: KeyHole satellite RS source image; b: KeyHole satellite RS image of the whole research area; c: Identification; d: Vector data).

KeyHole satellite RS images for 1961 were initially preprocessed. Image preprocessing included registration, enhancement, and cutting and splicing of the research area to generate an RS map. The polynomial correction method was used for graphic registration, while the 1980 land use status map was used as the basis for geometric correction and for the superposition of KeyHole satellite images. This land use map uses the GCS_Krasovsky_1940 coordinate system and so the data type is vector. Features that are easily distinguishable on the image, such as intersections in roads, river bends or bifurcations, were selected as control points which are distributed throughout the image. Images were enhanced using the contrast stretching method and information in the RS map and features of rural settlements were compared. Black and white KeyHole images show that rural settlements are clustered and are displayed in various colors as patched graphics closely linked by a number of small rectangles. Settlements are displayed in a white tone, interspersed with green vegetation and roads with mostly flat farmland are distributed in the vicinity of these locations (Fig. 1). These features were then used to create vector data for Tongzhou District rural settlements (Fig. 2).

### Result validation

Data for rural settlements in 1961 were verified using created and updated accurate evaluation points as well as a confusion matrix.

#### Accurate assessment points

Land use across the research area was divided into rural settlements and other land types. These were assigned values of 1 and 0, respectively, and were transformed into raster data types (named ru_classified). The ArcGIS 10.4 precision evaluation point tool was then used and each image was classified as an input layer based on ru_classified. The CLASSIFIED option was selected, and land use was assigned to each random point using a basic classification image. A total of 200 randomly distributed points were generated and the sampling scheme was EQUALIZED_STRATIFIED_RANDOM so that the number for each land use type remained the same. The point tool generated 200 vector layers formed by random points with each matching image classification information.

#### Updating accurate assessment points

We initially selected sample points for validation. These points were obtained through field research, or through the interpretation of higher precision images. It is obvious that we were not able to carry out field verification in 1961; no high precision image data are available for that year and so we used another reference method.

**Fig. 3:**
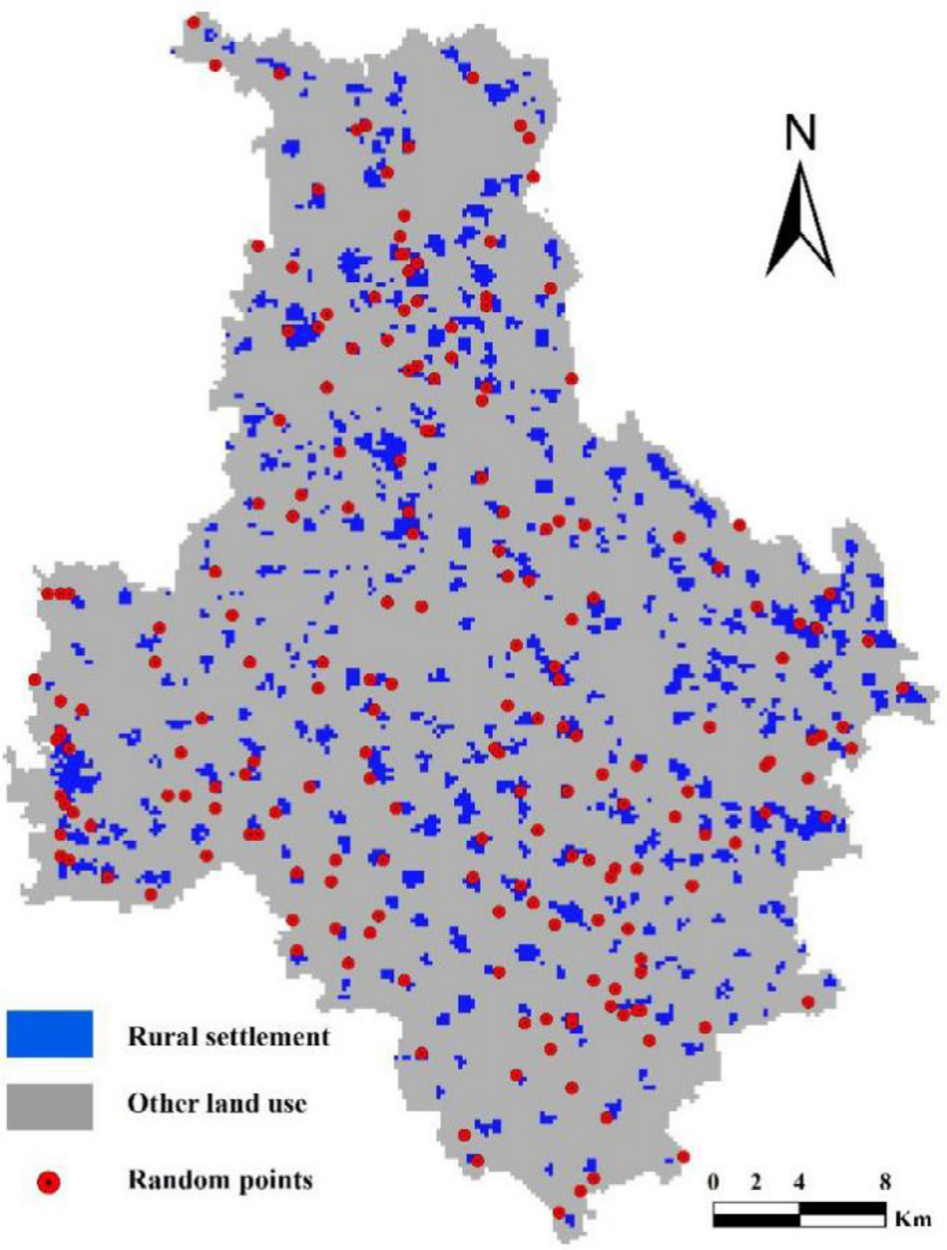
Map to show the distribution of 195 control points.

We used the imaging features of rural settlements and visual interpretation methods to identify these locations. This is an image interpretation method with relatively high accuracy, but one that may be subject to interpreter bias. Thus, in order to eliminate resultant uncertainty, we invited two other experts who are very experienced in image interpretation to jointly confirm our control points. These additional experts as well as the original image interpreters again identified the 200 classes of random points corresponding to ground classes. This means that when the results of all three people were identical, random points were identified as control points, while when results were inconsistent, we deleted random points. This approach enabled us to verify 195 control points (sample point layer referred to as point_refer) (Fig. 3).

**Fig. 4:**
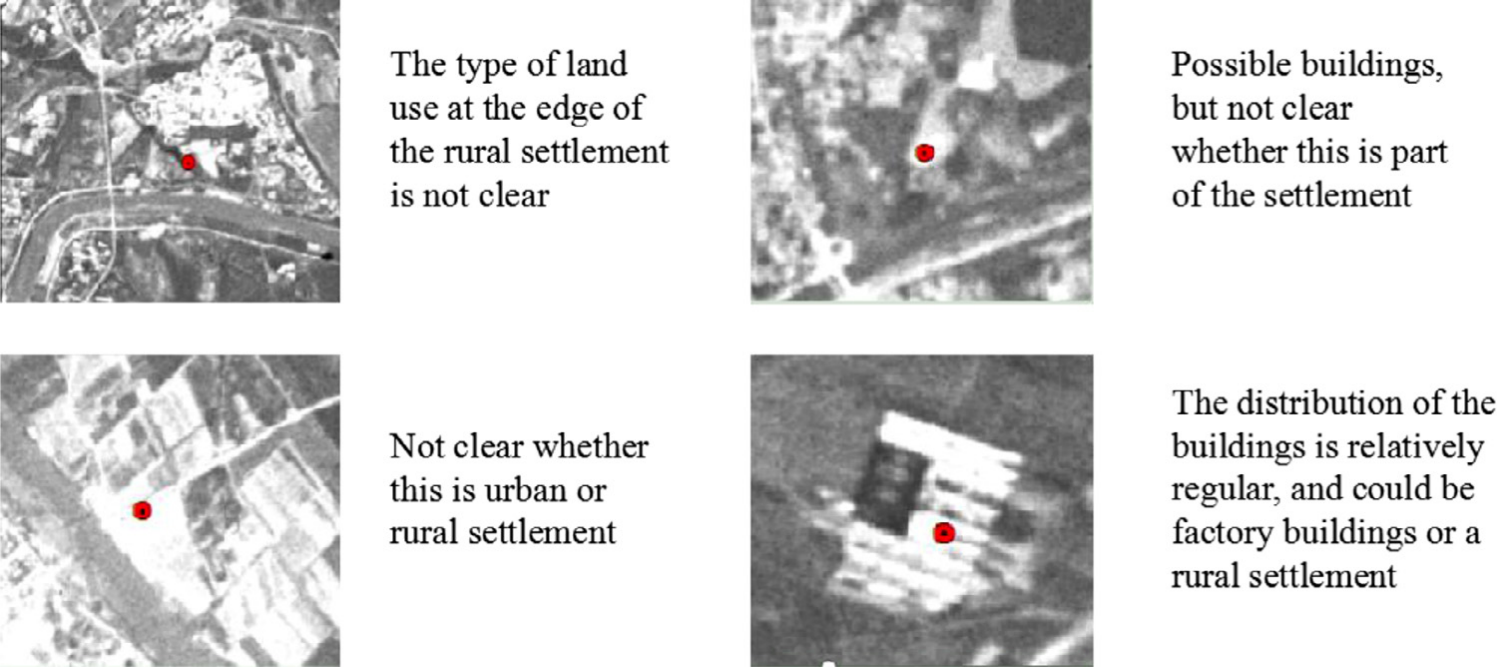
Objects corresponding to deleted points because of unrecognized land use types.

Three land class disagreements were identified when determining control points, mainly at boundaries between residential areas and other types of land use (Fig. 4).

**Fig. 5:**
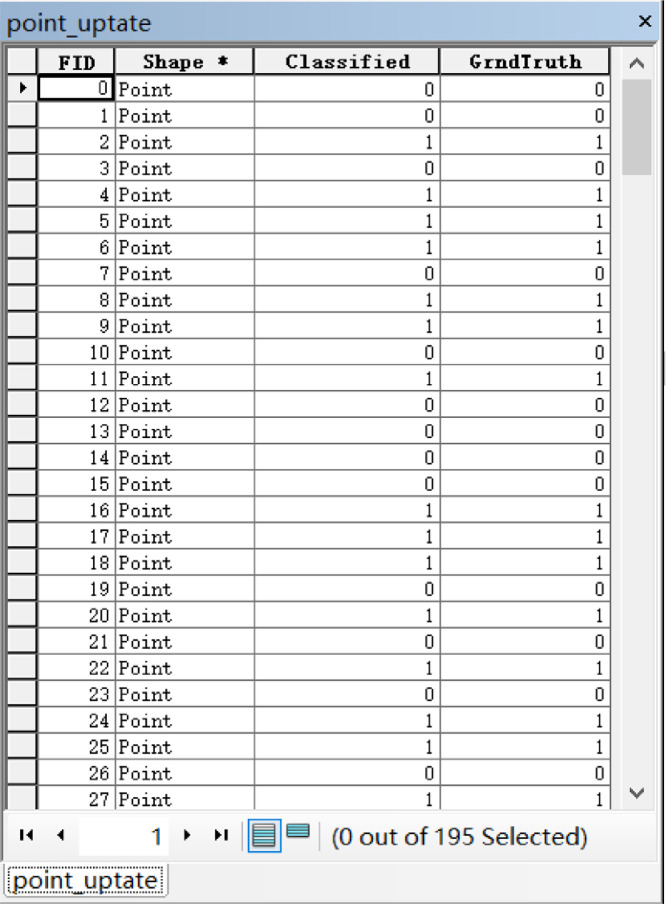
Example part of a point_update layer table (ArcGIS 10.4 display).

Actual land use corresponding to the 195 sample points was taken as input raster data using the ArcGIS 10.4 point tool. This means that the point_refer layer was used as input precision evaluation point data, the fields in the point_refer layer attribute table were updated, and the actual class information was updated to the point_refer layer. The point_update layer contains two fields, the Classified field which shows the classification result while the GrndTruth field displays actual land use information (Fig. 5).

#### Calculating a confusion matrix

The ArcGIS 10.4 computing confusion matrix tool was applied with a point_update layer used to evaluate input accuracy (Table 2).

**Table 2:** Rural settlement image interpretation results for 1961.

Class Value	0	1	Total	U_Accuracy	Kappa
0	96.00	0.00	96.00	1.00	0.00
1	3.00	96.00	99.00	0.97	0.00
Total	99.00	96.00	195.00	0.00	0.00
P_Accuracy	0.97	1.00	0.00	0.98	0.00
Kappa	0.00	0.00	0.00	0.00	0.97

Abbreviations: 1 denotes rural settlements while 0 indicates other categories, U_Accuracy denotes user accuracy, and P_Accuracy denotes producer accuracy.

## Rural settlement spatial data resampling

As the spatial resolution and geographic coordinates of rural settlements differ in 1961 from those of other years, data consistency and comparability were not uniform across the timescale of this analysis. Spatial data from rural settlements from different sources in different years were transformed into uniform geographic coordinates and uniform projection coordinates were defined.

Rural settlements in 1961 were identified using 2 m resolution KeyHole satellite images as source data. These points were transformed into raster data with a 30 m spatial resolution. This is consistent with the resolution of data for rural settlements in 1980, 2000, and 2015. Spatial coordinates of rural settlements raster data in 1961 are defined as the geographic and projection coordinate systems consistent with the spatial data of rural settlements in 1980, 2000, and 2015. This approach enables us to conduct an evolutionary analysis of rural settlements in different years under a unified coordinate system and with uniform data accuracy.

The method described in this paper can be used for the acquisition of high-precision spatial data about rural settlements.

## Declaration of Competing Interest

The authors declare that they have no known competing financial interests or personal relationships that could have appeared to influence the work reported in this paper.
